# Autoimmunity in IgA nephropathy

**DOI:** 10.3389/fimmu.2026.1782872

**Published:** 2026-03-09

**Authors:** Yoshihito Nihei, Yusuke Suzuki

**Affiliations:** Department of Nephrology, Juntendo University Faculty of Medicine, Bunkyo-ku, Tokyo, Japan

**Keywords:** autoantibody, autoimmune disease, autoimmunity, gd-IgA1, IgA nephropathy, IgA-MESCA, muti-hit hypothesis

## Abstract

IgA nephropathy (IgAN) is the most common primary glomerulonephritis worldwide. Its pathogenesis is widely described by a multi-hit hypothesis in which galactose-deficient IgA1 (Gd-IgA1) serves as a central disease driver. Advances in the understanding of IgAN pathophysiology, together with the establishment of proteinuria reduction as a surrogate endpoint in 2019, have led to revisions of recent KDIGO Clinical Practice Guideline. As of 2025, multiple therapeutic agents are under active development and in clinical trials, with several already approved, highlighting the need for individualized treatment strategies. Optimizing the use of these emerging therapies requires a deeper understanding of disease mechanisms. One of the key unresolved questions in pathogenesis of IgAN is why Gd-IgA1-containing immune complexes selectively deposit in the glomerular mesangial region, a disease hallmark of IgAN. Long before the identification of Gd-IgA1, it has been debated whether mesangial immune complex deposition reflects passive trapping of circulating complexes or active deposition mediated by antibodies recognizing glomerular antigens. In this regard, we recently discovered IgA-type anti–mesangial cell antibodies (IgA-MESCA) in serum from patients with IgAN and demonstrated that these antibodies target the mesangial cell–surface antigens β2-spectrin and CBX3. In this review, we summarize evidence from early studies to recent findings, including ours, on autoantibodies in IgAN, with a particular focus on glomerular-specific autoantibodies, and discuss the potential involvement of these autoantibodies in the pathogenesis of IgAN.

## Introduction

IgA nephropathy (IgAN) is characterized by predominant deposition of IgA antibody (Ab) in the mesangial regions of the glomeruli and represents the most common form of primary glomerulonephritis worldwide (1). Co-deposition of complement C3 is frequently observed (2, 3), whereas IgG or IgM co-deposition may also be present (4). Although epidemiological features vary by country and region, the prevalence has been reported to be approximately 2.5 per 100,000 adults (5), with a particularly high frequency in Asian populations (6, 7).

Analyses using the United Kingdom National Registry of Rare Kidney Diseases (RaDaR) (8), together with large cohort studies from China and Japan investigating the prognosis of more than 1,000 patients with IgAN (9, 10), have led to the conclusion that the long-term prognosis of IgAN is not necessarily favorable (11). Accordingly, the KDIGO 2025 Clinical Practice Guideline recommends individualized lifetime-based prognostic assessment in IgAN and initiation of therapy in patients with proteinuria ≥0.5 g/day, with treatment targets of reducing proteinuria to <0.5 g/day (ideally at <0.3 g/day) and limiting the annual eGFR decline to <1 mL/min/1.73 m² (11).

It is now well established that IgA deposited in the glomeruli of patients with IgAN consists predominantly of IgA1 carrying O-glycoforms with reduced galactose residues in the hinge region (12–14)—commonly reported as galactose-deficient IgA1 (Gd-IgA1) —and that circulating levels of Gd-IgA1 are increased in patients with IgAN (15, 16). These findings have led to broad acceptance of a “multi-hit hypothesis” in which Gd-IgA1 plays a central role in disease pathogenesis (17, 18).

Following the U.S. Food and Drug Administration’s acceptance of proteinuria reduction as a surrogate endpoint in IgAN in 2019, numerous novel therapeutic agents have been developed based on the multi-hit hypothesis and have entered clinical trials (19). As of 2025, more than ten novel therapeutic agents are under clinical development, with several having already demonstrated efficacy and been approved for clinical use (17, 20).

IgAN exhibits marked clinical and pathological heterogeneity, ranging from cases detected by asymptomatic hematuria and proteinuria to those presenting with gross hematuria or nephrotic syndrome (1). Determining how best to tailor emerging therapies to diverse disease phenotypes remains a major challenge. Addressing this requires a deeper understanding of IgAN pathophysiology; however, several key aspects of the disease mechanisms remain to be elucidated (11).

One of these unresolved aspects is the mechanism underlying the selective deposition of IgA in the glomerular mesangial region, a disease hallmark of IgAN. Although molecules such as CD89 and the transferrin receptor have been discussed as potential contributors to this deposition process (21–25), the multi-hit hypothesis does not fully account for the mechanism underlying selective IgA deposition in the mesangium (18).

In general, region-specific Ab deposition suggests the involvement of antigen–Ab interactions. From this perspective, it has long been debated whether immune complex (IC) deposition in the glomeruli reflects passive trapping of circulating complexes or, alternatively, active deposition driven by Ab recognition of target antigens expressed within the glomeruli. Accordingly, whether the ICs detected in IgAN contain Abs with specificity for glomerular structures—namely, autoantibodies (autoAbs)—represents a fundamental question for understanding IgAN pathophysiology.

In this review, we summarize evidence from early studies to recent findings on autoAbs in IgAN, with a particular focus on glomerular-specific autoAbs, discuss the potential involvement of these Abs in the pathogenesis of IgAN.

## AutoAbs against Gd-IgA1

The most widely recognized autoAbs in IgAN are IgG directed against Gd-IgA1. Tomana et al. demonstrated that sera from patients with IgAN contain increased levels of IgG Abs recognizing Gd-IgA1 (26, 27). To elucidate the molecular characteristics of anti–Gd-IgA1 IgG, Suzuki et al. established Epstein–Barr virus–immortalized IgG-secreting cell lines from lymphocytes derived from patients with IgAN and demonstrated that the secreted IgG exhibit alterations in the amino acid sequence of complementarity-determining region 3 within the variable region of the immunoglobulin heavy-chain gene (28). More recently, not only IgG but also IgM targeting Gd-IgA1 have been reported to be frequently detected in patients with IgAN (29).

Although IgG co-deposition in the glomeruli of IgAN has been considered not to be detectable in all patients (1), Rizk and Novak et al. demonstrated, using nanobody-based Abs with higher specificity and affinity than conventional immunofluorescence (IF) Abs, that IgG is in fact co-deposited in the glomeruli of all patients with IgAN (3, 30). Based on these reports, it is widely accepted that autoAbs recognizing Gd-IgA1 form ICs with Gd-IgA1 that deposit in the glomeruli and trigger inflammatory responses (27, 28, 30–32). Thus, IgAN is often described as an autoimmune disease in which IgA itself functions as an autoantigen (1, 33, 34). Details of IgG Abs against Gd-IgA1 have been described extensively elsewhere (35, 36).

## AutoAbs against glomerular components

A few years after the first description of IgAN, Lowance et al. demonstrated that IgA extracted from renal biopsy specimens of patients with IgAN contained IgA capable of binding to mesangial components of the normal kidney (37). This early study first proposed that IgA in IgAN may be directed toward glomerular mesangial components.

In the early 1980s, Tomino and colleagues conducted detailed investigations into the antigen specificity of IgA in patients with IgAN. They showed that IgA extracted from the glomeruli of IgAN patients bound to the glomerular mesangial region (38). It is interesting that they further reported that this extracted IgA reacted not only with mesangial regions but also with tonsillar cells (39). In addition, serum IgA from patients with IgAN was shown to bind to cultured fibroblasts (40). Although the target antigens were not identified in these studies, the findings collectively supported the idea that IgA in patients with IgAN may recognize certain autoantigens.

In addition to IgA, the presence of IgG-type autoAbs has also been reported in IgAN. Ballardie et al. investigated serum IgG against glomerular components in healthy individuals, patients with other forms of glomerulonephritis, and patients with IgAN. They detected IgG against glomerular components in 23 of 75 patients with IgAN, whereas such autoAbs were rarely observed in healthy controls or patients with other glomerular diseases. High-performance liquid chromatography demonstrated that this autoAbs recognizes multiple antigens. Moreover, IgAN patients positive for autoAbs exhibited a higher frequency of glomerular IgG deposition, and autoAb titers correlated with the degree of proteinuria. Although the specific target antigens recognized by these Abs were not identified in this study, the authors concluded that IgG directed against glomerular components may contribute to the pathogenesis of IgAN (41).

## AutoAbs against endothelial cells

In the late 1980s, growing interest was directed toward autoAbs against endothelial cells, referred to as anti-endothelial cell Abs (AECA). Yap and colleagues measured AECA in the sera of patients with IgAN and reported a significantly higher prevalence of AECA positivity in patients with IgAN compared with healthy controls (37% vs. 4%). Both IgA- and IgG-AECA were detected. In some patients, the presence of AECA was shown to be associated with the degree of proteinuria (42).

Wang et al. also examined the presence of AECA in the sera of patients with IgAN. According to their report, IgA-AECA was detected in 18 of 63 patients with IgAN (29%), whereas only 1 of 33 healthy controls (3%) was positive. Furthermore, Western blot analysis (WB) using endothelial cell membrane components revealed that IgA-AECA bound to proteins of approximately 135 kDa and 116 kDa in 6 patients (33%) and to a protein of approximately 205 kDa in 10 patients (56%). AECA levels were also shown to correlate with the degree of proteinuria and histological severity. However, IgA-AECA was also detected in 13 of 45 patients with lupus nephritis (29%). Therefore, Wang et al. suggested that although AECA cannot be considered specific to IgAN, they may act as disease-modifying factors that contribute to glomerular endothelial injury and exacerbate inflammation in IgAN (43).

## Collagen-binding IgA

The presence of IgA binding to glomerular basement membrane (GBM) extracts in patients with IgAN has also been discussed in early studies (44). Cederholm et al. reported that sera from patients with IgAN contained IgA that bound to human and bovine GBM extracts, particularly to the α chains of type IV collagen (45). Binding of IgA to collagen was preserved under reducing conditions but was abolished by collagenase treatment, leading the authors to conclude that serum IgA recognizes epitopes within the triple-helical domain of type IV collagen. These IgA Abs were shown to react not only with type IV collagen but also with type I and type II collagens. In subsequent studies, Cederholm et al. demonstrated that IgA binding, in fact, occur indirectly through IgA–fibronectin complexes via fibronectin–collagen interactions (46).

The group led by Jiří Mestecký also conducted detailed investigations into the possibility that IgA derived from patients with IgAN bind to extracellular matrix components produced by mesangial cells (47). Given that serum IgA levels are elevated in IgAN, Mestecký et al. selected patients with HIV infection exhibiting elevated IgA levels as controls and measured collagen-binding IgA in serum by ELISA. Using type I–VI collagens as antigens, they found that IgA binding to type I–VI collagens, except type IV collagen, was increased in sera from patients with IgAN. However, IgA binding to type II, type III, and type VI collagens was similarly increased in patients with HIV infection. Somewhat different from the findings of Cederholm et al, these results suggested that type IV collagen is not a major target of serum IgA in IgAN and that IgA binding to type II, type III, and type VI collagens does not explain mesangial IgA deposition in IgAN.

When sera were pretreated with gelatin which has fibronectin-binding domains before assessing IgA–collagen binding, IgA bindings to type I–IV collagens were reduced, whereas binding to type V and type VI collagens remained unchanged. Based on these findings, Mestecký et al. concluded that IgA binding to type V collagen may represent IgA-type autoAbs that directly target the collagen molecule itself, rather than indirect binding mediated by fibronectin. However, because type V collagen is widely expressed across multiple tissues, the authors pointed out a potential inconsistency: if such autoAbs contributed to disease pathogenesis, deposition would be expected in organs beyond the kidney.

## Anti-mesangial cell Abs

In the 1990s, the presence of autoAbs directed against mesangial cells was also investigated. Ballardie et al. discovered MESCA in the sera of patients with IgAN (48, 49). Using ELISA and WB with cultured mesangial cells, they demonstrated that IgG-MESCA binding to proteins of approximately 48 kDa and 55 kDa were specifically detected in the sera of patients with IgAN. Fornasieri et al. similarly confirmed the presence of IgG-MESCA in the sera of patients with IgAN (50).

Recently, we investigated the mechanism underlying the selective deposition of IgA-containing ICs in the mesangial region using the gddY mouse, a spontaneous model of IgAN (51, 52). The gddY mouse closely resembles human IgAN and shares IgAN susceptibility loci, including the human familial IGAN1 locus, indicating partially overlapping genetic determinants of disease (53, 54). On the basis of findings obtained from preclinical studies using this model (55–57), several international clinical trials have either been completed or are currently in progress (17, 58, 59).

Using IF staining and WB, we demonstrated the presence of IgA-MESCA in serum from gddY mice. Through immunoprecipitation using mouse serum or a mesangial cell–reactive monoclonal Ab established from IgA^+^ plasma cells of gddY mice, mass spectrometry, and WB–based screening, we identified β2-spectrin and CBX3 as their target autoantigens of IgA-MESCA. β2-spectrin is known to localize to the cytoplasm as a cytoskeletal protein (60), whereas CBX3 is a nuclear protein involved in heterochromatin formation (61). However, flow cytometry of mouse glomerular cells using Abs recognizing β2-spectrin and CBX3 revealed that both molecules are expressed on the cell surface exclusively by mesangial cells.

Anti–β2-spectrin IgA Abs and anti-CBX3 IgA Abs were not only detected in the sera of gddY mice but were also shown to be specifically present in the sera of patients with IgAN (62). Using two independent cohorts from Japan and the United Kingdom, serum levels of anti–β2-spectrin IgA Abs and anti-CBX3 IgA Abs were measured by ELISA in 119 patients with IgAN (70 Japanese and 49 British) and 51 patients with other kidney diseases (32 Japanese and 19 British).

Anti–β2-spectrin IgA Abs were positive in 15 of 70 Japanese patients with IgAN and in 15 of 49 British patients with IgAN. Among patients with other kidney diseases, no patients in the Japanese cohort were positive for anti–β2-spectrin IgA Abs, whereas 3 of 19 patients in the British cohort (1 with membranous nephropathy and 2 with ANCA-associated vasculitis) tested positive. Overall, anti–β2-spectrin IgA Abs were detected in 30 of 119 patients with IgAN and in 3 of 51 patients with other kidney diseases, corresponding to a sensitivity of 25% and a specificity of 94%.

In contrast, anti-CBX3 IgA Abs were detected in 25 of 70 Japanese patients with IgAN and in 23 of 49 British patients with IgAN. Among patients with other kidney diseases, positivity was observed in 1 of 32 Japanese patients (membranous nephropathy) and in 2 of 19 British patients (1 membranous nephropathy and 1 ANCA-associated vasculitis). Overall, anti-CBX3 IgA Abs were detected in 48 of 119 patients with IgAN and in 3 of 51 patients with other kidney diseases, yielding a sensitivity of 40% and a specificity of 94%.

Among Japanese patients with IgAN, 4 patients (6%) were positive for both anti–β2-spectrin IgA Abs and anti-CBX3 IgA Abs, whereas 32 patients (46%) were positive for either Ab. Among British patients with IgAN, 12 patients (25%) were positive for both Abs and 14 patients (29%) were positive for a single Ab. Overall, approximately half of patients with IgAN (52%) harbored IgA-MESCA recognizing β2-spectrin and/or CBX3 in their serum. Patients negative for both Abs may possess IgA-MESCA with specificity for other autoantigens.

**Figure 1:**
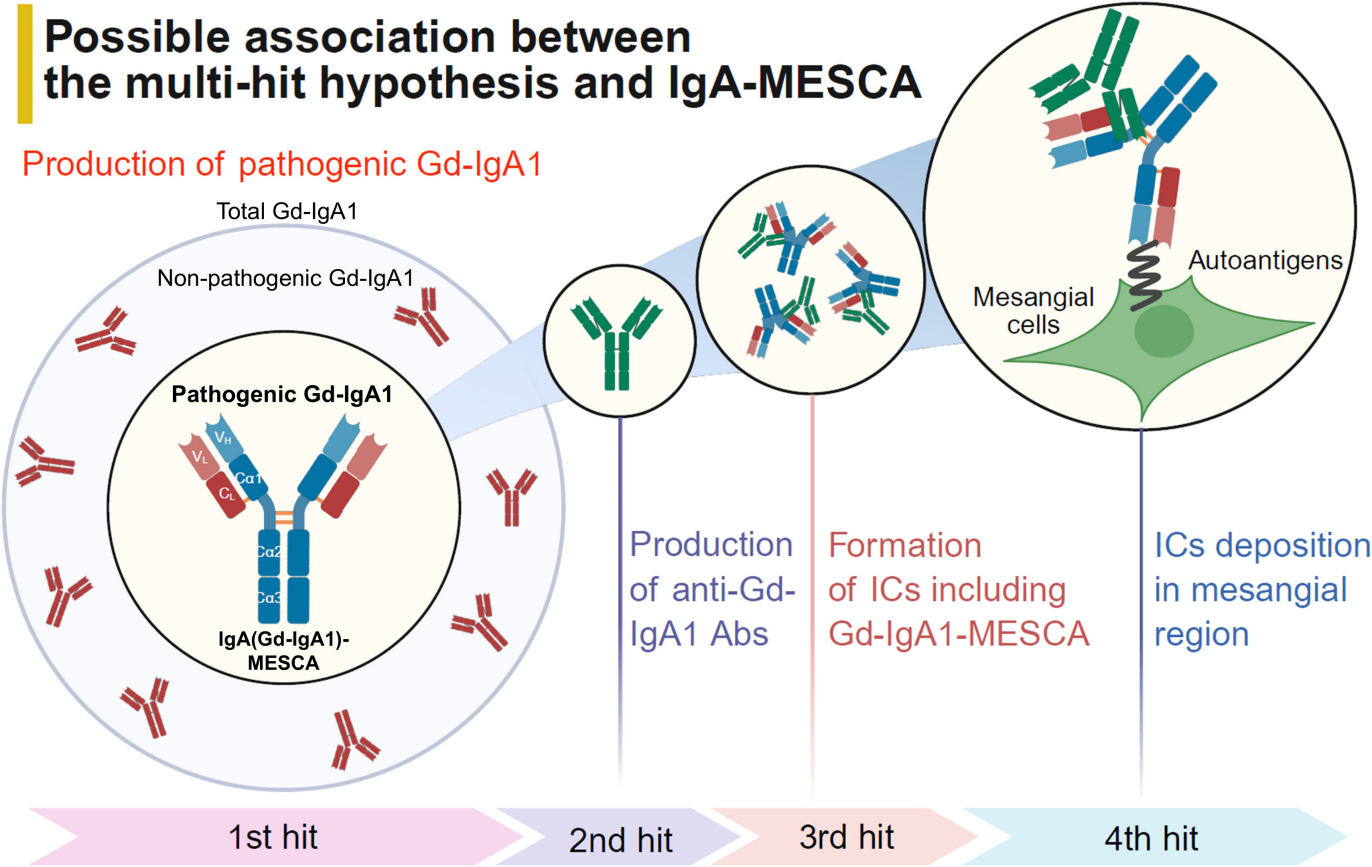
Possible association between the multi-hit hypothesis and IgA-MESCA. Only a small fraction of circulating Gd-IgA1 may be pathogenic. The discovery of Gd-IgA1-MESCA—an IgA-MESCA that targets mesangial cells and exhibits features of Gd-IgA1-provides a potential explanation for the mesangial deposition of Gd-IgA1–containing immune complexes within the multi-hit hypothesis. VH, valuable region of heavy chain; VL, valuable region of light chain; Cα, constant region of α chain; CL, constant region of light chain; ICs, immune complexes. Created in BioRender.com.

Interestingly, anti–β2-spectrin IgA Abs and anti-CBX3 IgA Abs isolated from the sera of patients with IgAN were recognized by KM55, a monoclonal Ab specific for Gd-IgA1 (63). This finding suggests that IgA-MESCA in patient serum present exhibit the characteristics of Gd-IgA1, indicating the presence of Gd-IgA1-MESCA, and may provide a potential explanation for mesangial deposition of Gd-IgA1 in the context of the multi-hit hypothesis (**Figure 1**).

The results of a phase II clinical trial of the B cell–directed therapeutic agent felzartamab, which targets the plasma cell surface marker CD38, were recently reported (64). In this trial, patients received felzartamab for 6 months, followed by an 18-month observation period. Notably, felzartamab treatment was associated with reductions in serum Gd-IgA1 levels accompanied by improvement in proteinuria; however, after treatment discontinuation, serum Gd-IgA1 levels rebounded, whereas the improvement in proteinuria was maintained. These findings suggest that only a small fraction of total circulating Gd-IgA1 in patient serum—designated as “pathogenic Gd-IgA1”—may actually contribute to the disease activity. From this perspective, our findings raise the possibility that Gd-IgA1-MESCA, which targets mesangial cells and possesses features of Gd-IgA1, may be relevant to the pathogenicity of Gd-IgA1 (**Figure 1**). However, at present, there is no direct evidence demonstrating that Gd-IgA1-MESCA contributes to the pathogenesis of IgAN; therefore, further investigation through both basic and clinical studies is warranted.

## Discussion

In this review, we provide an overview of the understanding of autoAbs in IgAN (**Table 1**). IgAN has been regarded as an autoimmune disease based on the presence of IgG autoAbs against Gd-IgA1; however, considering the studies reviewed here, IgAN is an IC-mediated disease and may, at the same time, be considered an autoimmune disease characterized by the presence of glomerular-specific autoAbs (65).

**Table 1:** Summary of autoAbs described in this review.

Target	Target autoantigens or sites	Subclass	Notes	Ref.
Gd-IgA1	GalNAc in the hinge region of Gd-IgA1	IgG, IgM, IgA	The most widely accepted autoAbs in IgAN	(3, 26–30)
			AutoAbs against Gd-IgA1 form ICs that deposit in the glomeruli.	
Glomerular components	Undetermined	IgA	Extracted glomerular IgA react both mesangial region and tonsillar cells.	(38–40)
			Serum IgA bind to cultured fibroblasts.	
	MW (kDa): 185, 160 and 30–40 kDa (HPLC), 69–30 kDa (deaggregated)	IgG	Patients positive for autoAbs exhibit a higher frequency of glomerular IgG deposition.	(41)
			AutoAb titers correlate with the degree of proteinuria.	
Endothelial cells	MW (kDa): 116 kDa, 135 kDa, and 205 kDa	IgA, IgG	AECA levels correlate with the degree of proteinuria and histological severity in some patients.	(42, 43)
			AECA is detected in approximately 30% of patients with lupus nephritis.	(43)
Collagen	Type V collagen	IgA	Type V collagen is widely expressed across multiple tissues.	(47)
Mesangial cell	MW (kDa): 48 kDa and 55 kDa	IgG	The episodes of nephritis are associated with high levels of circulating autoAbs.	(48–50)
	β2-spectrin, CBX3	IgA	Approximately half of IgAN patients is positive for IgA-MESCA.	(51, 52, 62)
			IgA-MESCA isolated from the sera of patients with IgAN is recognized by KM55.	(62)

Gd-IgA1, galactose-deficient IgA1; GalNAc, N-acetylgalactosamine; AutoAbs, autoantibodies; MW, molecular weight; HPLC, high-performance liquid chromatography; AECA, Abs directed against endothelial cells; MESCA, anti-mesangial cell Abs.

Lafayette et al. investigated the presence of autoAbs in the sera of patients with IgAN and demonstrated that IgAN patients harbor IgA- and/or IgG-type autoAbs against multiple antigens (66, 67). These findings indicate the presence of underlying immunological abnormalities in IgAN that predispose to autoAbs production (68).

Clarifying whether glomerular-specific autoAbs are directly involved in the pathogenesis of IgAN, how they relate to Gd-IgA1, and how such autoAbs are generated would deepen our understanding of disease mechanisms and may help guide the use of new therapeutic agents for IgAN in the future.
